# Shikonin protects against lipopolysaccharide‐induced inflammation and apoptosis in human nucleus pulposus cells through the nuclear factor‐kappa B pathway

**DOI:** 10.1002/fsn3.2519

**Published:** 2021-08-10

**Authors:** Yuanbin Liu, Jiazhuang Zheng, Yu Chen, Fandong Wang, He Ye, Miao Wang, Zhi Zhang

**Affiliations:** ^1^ Department of Orthopaedics Suining Central Hospital Suining China

**Keywords:** apoptosis, inflammation, NF‐κB pathway, nucleus pulposus cells, shikonin

## Abstract

**Objective:**

To investigate the protective effect and mechanism of shikonin on human intervertebral disk degeneration.

**Methods:**

Human primary nucleus pulposus (NP) cells cultured in vitro were used for the experiments. The effects of different concentrations of shikonin (1, 2, 4, 8, and 16 µM) on the activity of lipopolysaccharide (LPS)‐induced NP cells were determined using the CCK‐8 assay, and the appropriate drug concentration was determined. The experiment was divided into the control, LPS, and LPS + shikonin groups. ELISA and Western blot were used to detect the expression of the inflammatory factors tumor necrosis factor (TNF)‐α and interleukin (IL)‐1β. NP cell apoptosis was measured using Western blot and caspase 3 activity. Western blot and immunofluorescence assays were used to detect the protein expression of p‐P65 and P65 and the nuclear translocation of P65.

**Results:**

The CCK‐8 assay showed that shikonin had no cytotoxic effect on NP cells and increased the activity of LPS‐induced NP cells, especially at a concentration of 4 μM. Shikonin reversed the expression of the inflammatory cytokines TNF‐α and IL‐1β and apoptosis‐related molecules Bax, Bcl‐2, and cleaved caspase 3 in LPS‐induced NP cells. In addition, shikonin significantly decreased apoptosis and caspase‐3 activity in LPS‐induced NP cells. Furthermore, shikonin treatment significantly inhibited the expression of p‐P65 and nuclear translocation of P65, and nuclear factor‐kappa B (NF‐κB) pathway inhibitor Pyrrolidinedithiocarbamate ammonium (PDTC) significantly enhanced the anti‐inflammatory and antiapoptotic effects of shikonin in LPS‐induced NP cells.

**Conclusion:**

Shikonin significantly inhibited the inflammatory response and apoptosis of human primary NP cells, possibly through the NF‐κB pathway.

## Graphical abstract

Shikonin can significantly inhibit the inflammatory response and apoptosis of human intervertebral disk nucleus pulposus cells induced by lipopolysaccharide, and the mechanism may be through the nuclear factor‐kappa B pathway. The current research results may help to identify the theoretical basis for future research on the potential therapeutic effects of shikonin in the clinical treatment of preventing degenerative changes of intervertebral discs.

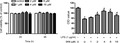

## 1 INTRODUCTION

Intervertebral disk degeneration (IVDD) is the initiating factor of spinal degenerative diseases and is one of the main causes of chronic low‐back pain. Its prevalence is increasing, which has caused a serious economic and social burden (Cheung, 2010; Joud et al., 2012). Although the causes and molecular mechanisms of IVDD have not yet been fully elucidated, increased inflammation and excessive apoptosis of nucleus pulposus (NP) cells are important risk factors in the process of IVDD (Adams & Roughley, 2006; Ding et al., 2013; Wang et al., 2013). It has been reported that some cytokines, such as tumor necrosis factor (TNF)‐α and interleukin‐1β (IL‐1β), can significantly decrease the gene and protein levels of aggrecan and collagen II, and increase the gene and protein levels of matrix metalloproteinases (MMPs) and a disintegrin and metalloproteinase with thrombospondin motifs, thereby leading to the degradation of IVDD (Johnson et al., 2015; Wang, Che, et al., 2020). Some studies have showed that the decreased number of NP cells caused by their excessive apoptosis leads to a reduction in extracellular matrix synthesis, which further leads to disk degeneration (Ding et al., 2013; Luo et al., 2019;). As a result, finding a suitable treatment that can inhibit the inflammatory response and excessive apoptosis of NP cells may delay intervertebral disk degeneration.

Nuclear factor‐kappa B (NF‐κB) is a dimer complex composed of p65/p50, which regulates cell apoptosis and inflammatory responses and participates in cell proliferation, differentiation, and immune response. The NF‐κB pathway is closely associated with IVDD. Shen et al. (2016) found that overexpression sirtuin 1 (SIRT1) protects against NP cell apoptosis through the NF‐κB pathway. Yi et al. (2019) also found that inhibiting NF‐κB can reduce the expression of inflammatory factors and cell apoptosis in IVDD. These results indicate that the NF‐κB pathway plays a vital role in delaying IVDD. Therefore, finding appropriate drugs that act on the NF‐κB pathway can potentially delay IVDD.

Shikonin is an active naphthoquinone extracted from the traditional Chinese medicinal herb, Lithospermum erythrorhizon, which is widely used because of its various pharmacological properties, such as antitumor, antioxidation, anti‐inflammatory, antiapoptotic, and wound‐healing properties (Guo et al., 2019; Imai et al., 2019; Lu et al., 2011; Wang, Mayca Pozo, et al., 2020; Yang et al., 2017). Previous research has shown that shikonin inhibits inflammation and chondrocyte apoptosis by regulating the PI3K/Akt signaling pathway in a rat model of osteoarthritis (Fu et al., 2016). In addition, shikonin exerts anti‐inflammatory effects in lipopolysaccharide (LPS)‐induced mastitis by inhibiting the NF‐κB signaling pathway (Yang et al., 2018). However, the role and molecular mechanism of action of shikonin in human IVDD remains unclear. Therefore, we explored whether shikonin exerts anti‐inflammatory and antiapoptotic effects through the NF‐κB pathway in LPS‐induced human NP cells.

## 2 MATERIALS AND METHODS

### 2.1 Patient tissue samples

Human disk tissues were derived from 12 patients (five women and seven men, aged 48–68 years) who underwent spinal surgery for lumbar disk herniation in our department. The collected intervertebral disk degeneration samples were determined into grades III to V according to the Pfirrmann grading scale (Pfirrmann et al., 2001). Written informed consent was obtained from all patients, and this study was approved by the Ethics Committee of Suining Central Hospital.

### 2.2 Cell isolation and culture

The disk tissues were rinsed several times with phosphate‐buffered saline (PBS) solution, cut into 1 mm^3^ pieces, and 2.5 g/L trypsin was added for 30 min. Then, the supernatant was centrifuged at 800 rpm for 5 min, and 2 g/L type II collagenase was added for 4 h. Next, 200 mesh cell sieves were used to filter the digested tissue. After centrifugation, the cell pellet was collected. Finally, the cells were cultured in Dulbecco's modified Eagle medium (DMEM)/F12 medium containing 10% fetal bovine serum (FBS) and 100 U/ml penicillin at 37°C in a 5% CO_2_ environment. Third‐generation NP cells were used for all experiments.

### 2.3 Cell viability assay

The third‐generation NP cells were digested with 0.25% trypsin and then seeded at a density of 1.5 × 10^4^ cells/ml in a 96‐well culture plate for 24 h. The cells were then cultured with 100 µM LPS and different concentrations (0, 1, 2, 4, 8, and 16 µM) of shikonin (Sigma‐Aldrich). After 24 h of incubation, 10 µl of CCK‐8 reagent was added to each well for 2 h. The optical density was measured at a wavelength of 450 nm using a Bio‐Rad 680 microplate reader. Each experiment was performed in triplicates.

### 2.4 ELISA

After NP cells were treated for 24 h, the cell supernatants from each group were collected. The expression of inflammatory factors (TNF‐α and IL‐1β) was detected using ELISA kits (Elascience) according to the manufacturer's instructions.

### 2.5 Hoechst 33258 staining

According to the results of the CCK‐8 assay, the experiment was divided into control group, LPS (1 µg/ml) and shikonin (4 µM) + LPS (1 µg/ml) groups. NP cells were seeded in a 24‐well plate at a density of 2 × 10^4^/well. After 24 h of treatment, NP cells were fixed with 4% paraformaldehyde for 15 min, followed by staining with 2 μg/ml Hoechst 33258 for 10 min at 37°C. Apoptotic cells were characterized by bright fluorescent DNA fragmentation and chromatin condensation under fluorescence microscopy.

### 2.6 Caspase 3 activity

Nucleus pulposus cells were seeded at 1 × 10^5^ cells/ml in six‐well plates and treated with the caspase tetrapeptide substrate Ac‐DEVD‐pNA (Beyotime) to detect caspase 3 activity. Cells were washed with PBS and lysed with 100 μl of lysis buffer (Beyotime) on ice for 15 min. The cells were incubated in a mixture of 10 μl lysate, 80 μl reaction buffer, and 10 μl AC‐DEVD‐PNA at 37°C for 1 h. The optical density of the free PNA was determined using a microplate spectrophotometer.

### 2.7 Immunofluorescence

The cells in each group were fixed with 4% paraformaldehyde for 10 min and then washed with PBS. The NP cells were permeabilized with 0.5% Triton for 3 min and then blocked with 0.1% BSA for 1 h. The p65 antibody dilution solution was added to each group and incubated at 4°C overnight. The appropriate diluted secondary antibody was added at room temperature for 2 h then, DAPI staining solution was added at room temperature for 10 min and the cells were observed under a fluorescence microscope.

### 2.8 Western blot

After treatment for 24 h, the cells were washed repeatedly with precooled PBS and lysed with RIPA lysis buffer on ice for 40 min. The cells were centrifuged for 15 min, and the supernatant was collected. The protein concentration was determined using a protein assay kit (Beyotime, P0010S). The protein was loaded to an SDS‐PAGE gel for electrophoresis and then transferred to a 0.22 µM PVDF membrane. After blocking at room temperature for 2 h, the membrane was incubated with the primary antibodies (anti‐TNF‐α, BA0131, Boster; anti‐IL‐1β, A00101, Boster; anti‐p‐P65, #3033, CST; anti‐P65, #8242, CST; anti‐cleave caspase 3, #9664, CST; anti‐Bax, TA810334, OriGene; and anti‐Bcl‐2, TA806591, OriGene) at 4°C overnight. The membrane was incubated with the corresponding secondary antibody for 2 h at room temperature. Finally, the membranes were measured using an ECL Plus reagent (Millipore).

### 2.9 Statistical analysis

SPSS 20.0 software was used for statistical analysis. The experimental data are expressed as the mean ± *SD* and analyzed by one‐way ANOVA and Student's *t*‐test. A *p* value <.05 was considered significant.

## 3 RESULTS

### 3.1 Effect of shikonin on the activity of NP cells induced by LPS

**FIGURE 1:**
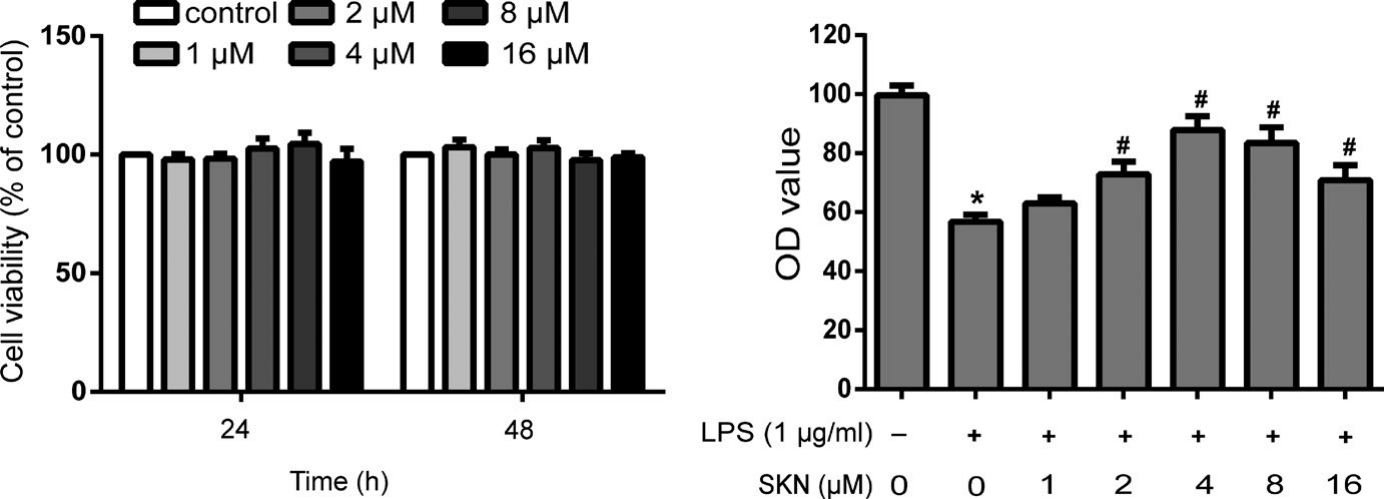
Effect of shikonin on the activity of nucleus pulposus cells induced by lipopolysaccharide (LPS). *n* = 3. All data were obtained from three independent experiments and shown as the mean ± *SD*. *p < .05, compared with control group, ^#^ *p* < .05, compared with LPS group

To investigate the cytotoxic effect of shikonin on NP cells, a CCK‐8 assay was used to detect NP cell viability. The results showed that shikonin had no significant effect on the activity of NP cells at 24 and 48 h after treatment with different concentrations (1, 2, 4, 8, and 16 μM), indicating that shikonin had no cytotoxic effect on NP cells. When NP cells were treated with LPS alone, the activity of NP cells decreased significantly (*p* < .05). After treatment with different concentrations of shikonin, the activity of NP cells increased to a certain extent compared to that of the LPS group. The activity of NP cells was highest when the concentration of shikonin was 4 μM. Therefore, we chose a shikonin concentration of 4 μM for the follow‐up experiment (Figure 1).

### 3.2 Shikonin inhibits the production of inflammatory cytokines in LPS‐induced NP cells

**FIGURE 2:**
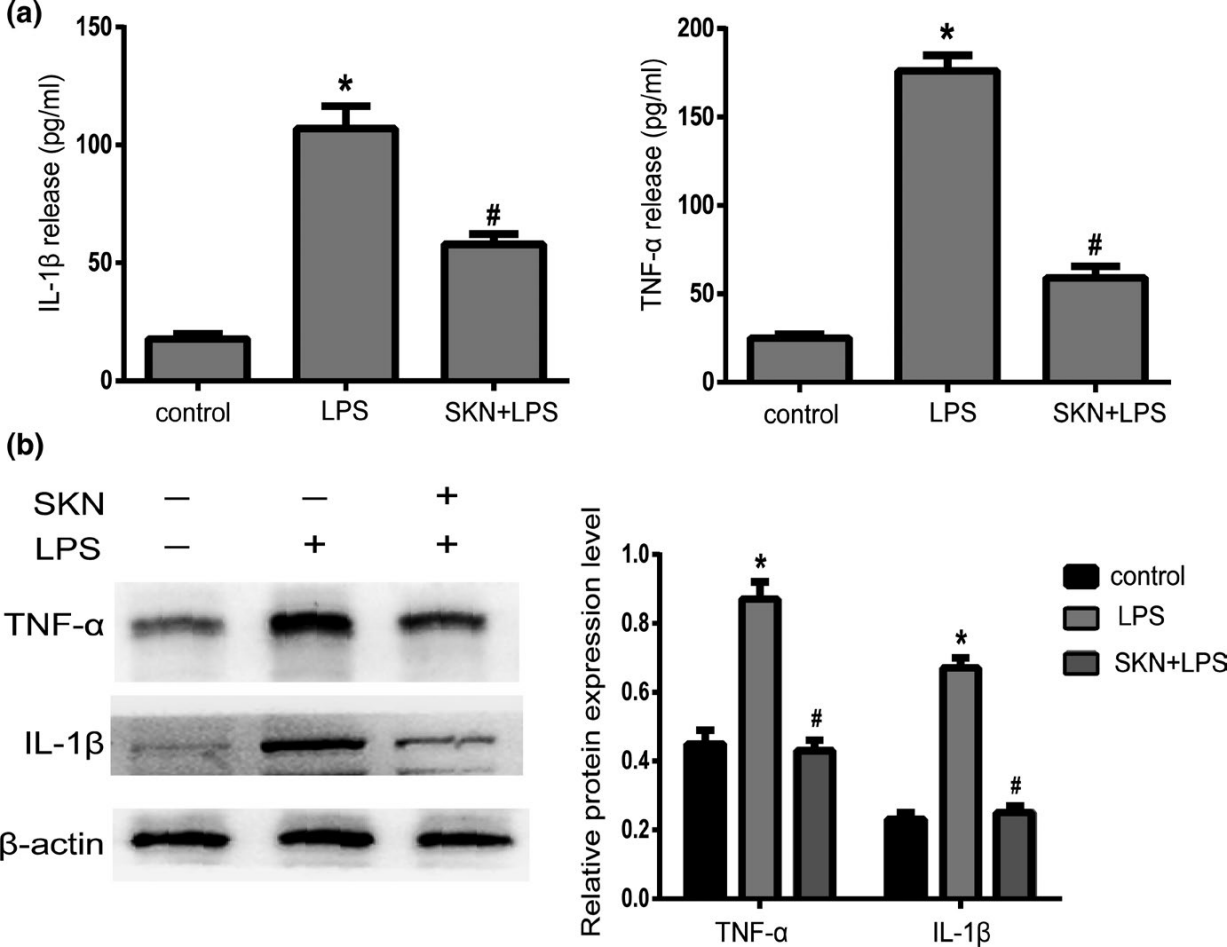
Shikonin inhibits the production of inflammatory cytokines in nucleus pulposus cells. (a) The expression of TNF‐α and IL‐1β in NP cells supernatant measured by ELISA, *n* = 3. (b) The protein expression of TNF‐α and IL‐1β detected by Western blot and relative expression of protein level, *n* = 3. All data were obtained from three independent experiments and shown as the mean ± *SD*. *p < .05, compared with control group, ^#^ *p* < .05, compared with lipopolysaccharide (LPS) group

The results of ELISA (Figure 2a) and Western blot (Figure 2b) show that the protein expression levels of TNF‐α and IL‐1β were lower in the control group. After treatment with LPS for 24 h, the protein expression levels of TNF‐α and IL‐1β were significantly increased (*p* < .05), but the protein expression levels of TNF‐α and IL‐1β were significantly decreased after shikonin treatment (*p* < .05).

### 3.3 Shikonin inhibits LPS‐induced apoptosis in NP cells

**FIGURE 3:**
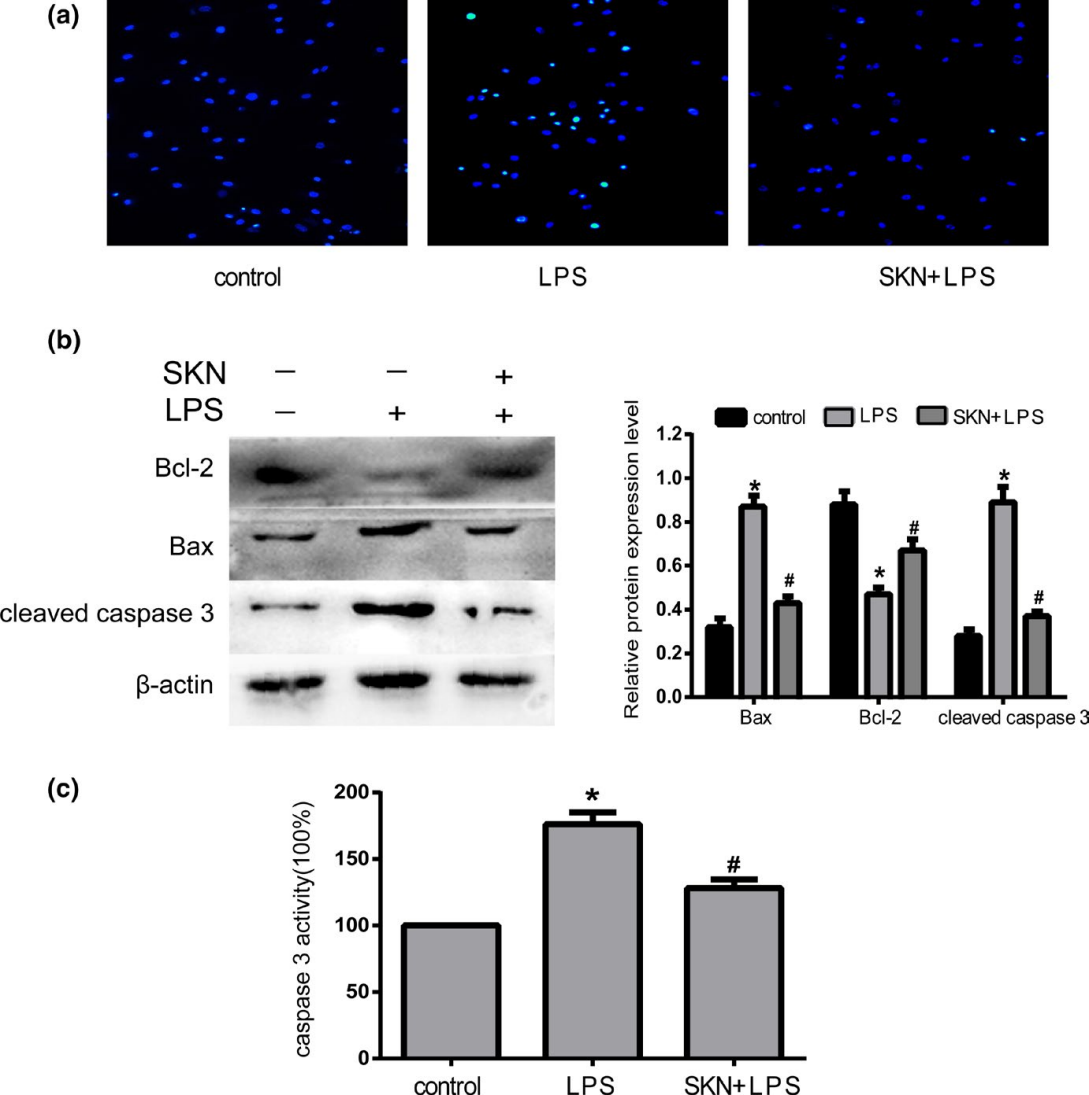
Shikonin inhibits lipopolysaccharide (LPS)‐induced nucleus pulposus cells apoptosis. (a) Morphologic changes in apoptotic NP cells were stained by Hoechst 33258, *n* = 3. (b) Expression of Bax, Bcl‐2 and cleaved caspase 3 detected by Western blot and relative expression of protein level, *n* = 3. (c) Detection of caspase 3 activity, *n* = 4. All data were obtained from three independent experiments and shown as the mean ± *SD*. *p < .05, compared with control group, ^#^ *p* < .05, compared with LPS group

Hoechst 33,58 staining showed that there were fewer apoptotic cells with highly bright fluorescent nuclei in the control and LPS + shikonin groups than in the LPS group (Figure 3a). Similarly, Western blot results showed that compared to the control group, the expression levels of Bax and cleaved caspase 3 were increased and the expression levels of Bcl‐2 were decreased after LPS treatment alone (*p* < .05). When shikonin was added, the expression levels of Bax and cleaved caspase 3 were decreased, and the expression levels of Bcl‐2 were significantly increased (*p* < .05; Figure 3b). Furthermore, the optical density value of the LPS group was significantly higher than that of the control group (*p* < .05), which indicated that LPS could increase caspase 3 activity. Meanwhile, after treatment with shikonin, the value of the LPS + shikonin group was significantly lower than that of the LPS group (*p* < .05; Figure 3c).

### 3.4 Shikonin inhibits the activation of NF‐κB pathway in LPS‐induced NP cells

**FIGURE 4:**
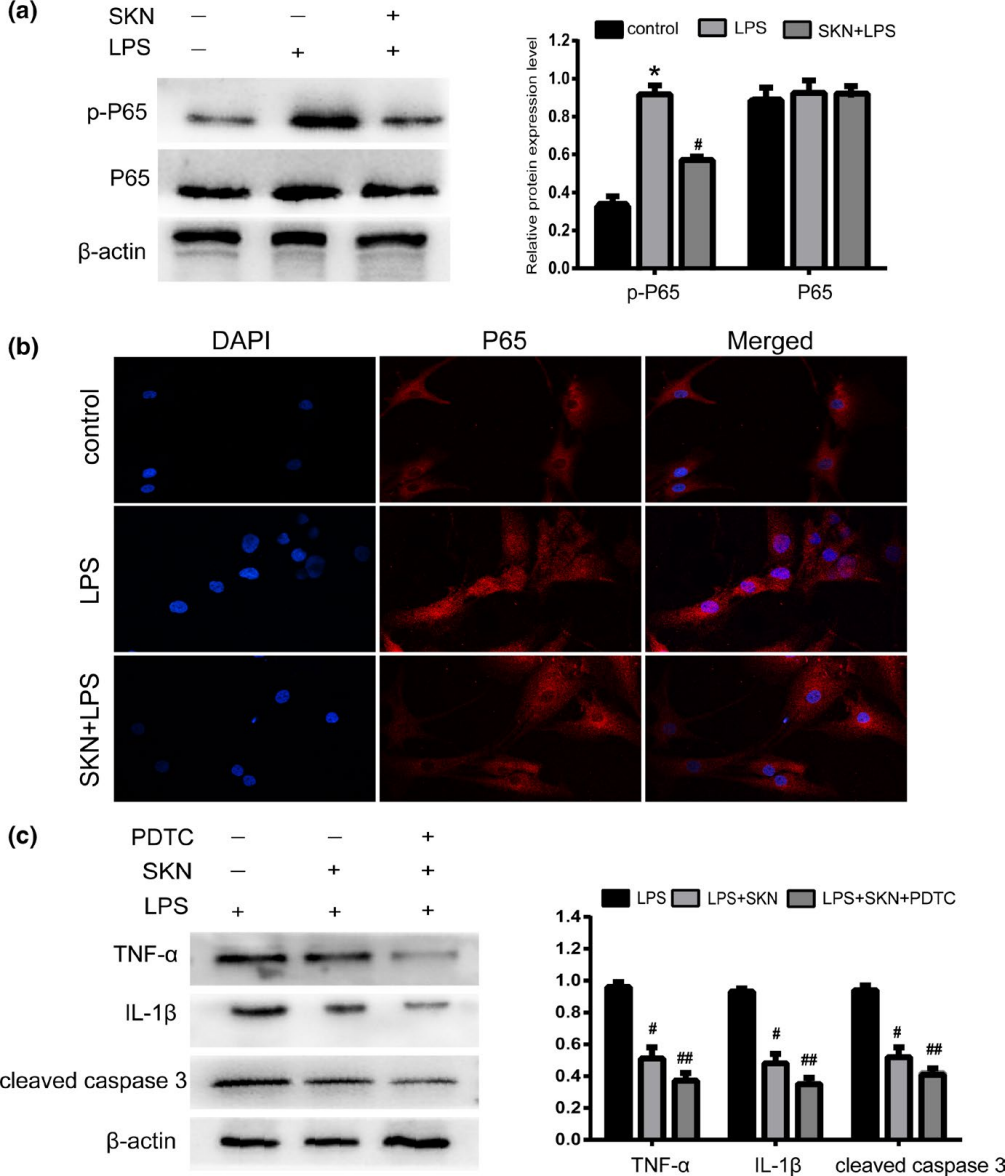
Shikonin inhibits the activation of nuclear factor‐kappa B (NF‐κB) pathway in lipopolysaccharide (LPS)‐induced nucleus pulposus (nucleus pulposus) cells. (a) The expression of p‐P65 and P65 proteins detected by Western blot and relative expression of protein, *n* = 4. (b) The nuclear translocation of P65 measured by immunofluorescence, *n* = 3 (magnification: ×200). (c) The expression of TNF‐α, IL‐1β, and cleaved caspase‐3 detected by Western blot and relative expression of protein, *n* = 4. All data were obtained from three independent experiments and shown as the mean ± *SD*. *p < .05, compared with control group, ^#^ *p* < .05, compared with LPS group, ^##^ *p* < .05, compared with LPS + SKN group

The experimental results showed that after treatment with LPS alone, the protein expression of p‐P65 in NP cells was significantly increased and the translocation of P65 to the nucleus was significant. After treatment with shikonin, p‐P65 protein expression in NP cells was significantly decreased (*p* < .05), and the nuclear translocation of P65 was significantly reduced. There was no significant difference in P65 protein expression between any of the groups (*p* > .05; Figure 4a,b). To further investigate whether NF‐κB signaling plays a role in LPS‐induced inflammation and apoptosis of NP cells, we used PDTC to inhibit NF‐κB signaling way. We found that NF‐κB signaling pathway inhibitor (PDTC) significantly enhanced the anti‐inflammatory and antiapoptotic effects of shikonin in LPS‐induced NP cells (Figure 4c).

## 4 DISCUSSION

Intervertebral disk degeneration is a multicausal age‐related degenerative disorder. Although the mechanism of disk degeneration is not completely clear, inflammation and excessive apoptosis are the main pathological factors of the process of disk degeneration (Liao et al., 2019; Risbud & Shapiro, 2014; Ruiz‐Fernandez et al., 2019). Shikonin has numerous pharmacological properties, including anti‐cancer, anti‐inflammatory, and antiapoptotic properties. However, the effect of shikonin on intervertebral disk degeneration has not yet been reported. Our results showed that pretreatment with shikonin inhibited cell inflammation and apoptosis in LPS‐induced human primary NP cells. In addition, shikonin increased p‐P65 expression and promoted nuclear translocation of P65. These results indicate that shikonin protects against LPS‐induced inflammation and apoptosis in human NP cells, possibly through the NF‐κB pathway.

Lipopolysaccharide, an admitted strong promoter of inflammation, can induce the production of inflammatory factors in NP cells and cause cell apoptosis, thereby reducing the content of collagen II and aggrecan, and ultimately leading to IVD degeneration (Dong et al., 2018; Zhong et al., 2021). Thus, in the present study, we used LPS to simulate an NP cell model of inflammation and apoptosis in degenerative intervertebral disks. Shikonin has anti‐inflammatory and antiapoptotic effects. A previous report indicated that shikonin attenuates hyperhomocysteinemia‐induced activation of CD4^+^ T cells in ApoE^−/−^ mice (Lu et al., 2020). Furthermore, recent reports pointed out that shikonin protects PC12 cells against β‐amyloid peptide‐induced cell injury through antioxidant and antiapoptotic activities (Tong et al., 2018). Our results also showed that LPS treatment significantly increased the expression levels of the inflammatory factors, TNF‐α and IL‐1β, and apoptosis in NP cells. However, the levels of inflammatory cytokines, TNF‐α and IL‐1β, and apoptosis decreased significantly in the NP cells after shikonin treatment. The above results indicate that shikonin possesses anti‐inflammatory and antiapoptotic roles in LPS‐induced NP cells, but the specific mechanism is not clear.

Nuclear factor‐κB is a dimer complex composed of p65/p50 and regulates cell apoptosis and the inflammatory response, and participates in cell proliferation, differentiation, and immune response. The NF‐κB pathway plays an important role in the degeneration of intervertebral disks (Dell'accio et al., 2008; Kang et al., 2017; Liang et al., 2018). Studies have reported that inhibiting the NF‐κB pathway can reduce the inflammatory response and cell apoptosis in LPS‐induced NP cells (Yi et al., 2019). Studies have confirmed that shikonin reduces edema induced by phorbol ester by interfering with IkappaBalpha degradation, thus inhibiting translocation of P65 to the nucleus, and shikonin suppresses osteoclastogenesis by inhibiting the NF‐κB pathway induced by RANKL (Andujar et al., 2010; Chen et al., 2020). However, the relationship between shikonin and the NF‐κB pathway in IVDD remains unclear. Our results showed that after treatment with LPS alone, the protein expression of p‐P65 and the nuclear translocation of P65 were significantly increased. However, shikonin treatment significantly decreased p‐P65 protein expression and the nuclear translocation of P65, NF‐κB signaling pathway inhibitor (PDTC) significantly enhanced the anti‐inflammatory and antiapoptotic effects of shikonin in LPS‐induced NP cells. These results indicated that the anti‐inflammatory and antiapoptotic effects of shikonin in LPS‐induced NP cells may be through inhibiting the NF‐κB pathway.

In conclusion, shikonin can significantly inhibit LPS‐induced inflammatory response and apoptosis in human NP cells, and the mechanism may be through the NF‐κB pathway. The results of the current study may help identify the theoretical basis for future research on the potential therapeutic effects of shikonin in the clinical treatment of preventing degenerative changes in intervertebral disks.

## CONFLICT OF INTEREST

The authors declare that there is no conflict of interest.

## AUTHOR CONTRIBUTIONS

**Yuanbin Liu:** Conceptualization (equal); Data curation (equal); Formal analysis (equal); Methodology (equal); Writing‐original draft (equal); Writing‐review & editing (equal). **Jiazhuang Zheng:** Conceptualization (equal); Methodology (equal); Resources (equal); Supervision (equal); Validation (equal); Writing‐review & editing (equal). **Yu Chen:** Data curation (equal); Formal analysis (equal); Methodology (equal); Resources (equal); Writing‐review & editing (equal). **Fandong Wang:** Data curation (equal); Methodology (equal); Resources (equal); Software (equal). **He Ye :** Formal analysis (equal); Software (equal); Writing‐review & editing (equal). **Miao Wang:** Formal analysis (equal); Software (equal); Writing‐review & editing (equal). **Zhi Zhang:** Formal analysis (equal); Funding acquisition (equal); Project administration (equal); Resources (equal); Supervision (equal); Writing‐original draft (equal); Writing‐review & editing (equal).

## ETHICAL APPROVAL

Informed consent was obtained from all individual participants included in the study, and the study protocol was subject to approval by the Ethics Committee of Suining Central Hospital.

## Data Availability

The data that support the findings of this study are available from the corresponding author upon reasonable request.

## References

[fsn32519-bib-0001] Adams, M. A. , & Roughley, P. J. (2006). What is intervertebral disc degeneration, and what causes it? Spine, 31(18), 2151‐2161. 16915105 10.1097/01.brs.0000231761.73859.2c

[fsn32519-bib-0002] Andujar, I. , Recio, M. C. , Bacelli, T. , Giner, R. M. , & Rios, J. L. (2010). Shikonin reduces oedema induced by phorbol ester by interfering with IkappaBalpha degradation thus inhibiting translocation of NF‐kappaB to the nucleus. British Journal of Pharmacology, 160(2), 376–388. 20423347 10.1111/j.1476-5381.2010.00696.x PMC2874859

[fsn32519-bib-0003] Chen, K. , Yan, Z. , Wang, Y. , Yang, Y. , Cai, M. , Huang, C. , Li, B. , Yang, M. , Zhou, X. , Wei, X. , Yang, C. , Chen, Z. , Zhai, X. , & Li, M. (2020). Shikonin mitigates ovariectomy‐induced bone loss and RANKL‐induced osteoclastogenesis via TRAF6‐mediated signaling pathways. Biomedicine & Pharmacotherapy, 126, 110067. 10.1016/j.biopha.2020.110067 32272431

[fsn32519-bib-0004] Cheung, K. M. (2010). The relationship between disc degeneration, low back pain, and human pain genetics. Spine Journal, 10(11), 958–960. 10.1016/j.spinee.2010.09.011 20970736

[fsn32519-bib-0005] Dell'accio, F. , De Bari, C. , Eltawil, N. M. , Vanhummelen, P. , & Pitzalis, C. (2008). Identification of the molecular response of articular cartilage to injury, by microarray screening: Wnt‐16 expression and signaling after injury and in osteoarthritis. Arthritis and Rheumatism, 58(5), 1410–1421. 10.1002/art.23444 18438861

[fsn32519-bib-0006] Ding, F. , Shao, Z. W. , & Xiong, L. M. (2013). Cell death in intervertebral disc degeneration. Apoptosis, 18(7), 777–785. 10.1007/s10495-013-0839-1 23512131

[fsn32519-bib-0007] Dong, Y. , Liu, L. , Shan, X. , Tang, J. , Xia, B. , Cheng, X. , Chen, Y. , & Tao, W. (2018). Pilose antler peptide attenuates LPS‐induced inflammatory reaction. International Journal of Biological Macromolecules, 108, 272–276. 10.1016/j.ijbiomac.2017.11.176 29208559

[fsn32519-bib-0008] Fu, D. , Shang, X. , Ni, Z. , & Shi, G. (2016). Shikonin inhibits inflammation and chondrocyte apoptosis by regulation of the PI3K/Akt signaling pathway in a rat model of osteoarthritis. Experimental and Therapeutic Medicine, 12(4), 2735–2740. 10.3892/etm.2016.3642 27703516 PMC5038895

[fsn32519-bib-0009] Guo, H. , Sun, J. , Li, D. , Hu, Y. , Yu, X. , Hua, H. , Jing, X. , Chen, F. , Jia, Z. , & Xu, J. (2019). Shikonin attenuates acetaminophen‐induced acute liver injury via inhibition of oxidative stress and inflammation. Biomedicine & Pharmacotherapy, 112, 108704. 10.1016/j.biopha.2019.108704 30818140

[fsn32519-bib-0010] Imai, K. , Kato, H. , Taguchi, Y. , & Umeda, M. (2019). Biological effects of shikonin in human gingival fibroblasts via ERK 1/2 signaling pathway. Molecules, 24(19), 3542. 10.3390/molecules24193542 PMC6804247 31574951

[fsn32519-bib-0011] Johnson, Z. I. , Schoepflin, Z. R. , Choi, H. , Shapiro, I. M. , & Risbud, M. V. (2015). Disc in flames: Roles of TNF‐alpha and IL‐1beta in intervertebral disc degeneration. European Cells & Materials, 30, 104–116, discussion 116–107. 26388614 10.22203/ecm.v030a08 PMC4751407

[fsn32519-bib-0012] Joud, A. , Petersson, I. F. , & Englund, M. (2012). Low back pain: Epidemiology of consultations. Arthritis Care & Research, 64(7), 1084–1088. 10.1002/acr.21642 22337573

[fsn32519-bib-0013] Kang, L. , Hu, J. , Weng, Y. , Jia, J. , & Zhang, Y. (2017). Sirtuin 6 prevents matrix degradation through inhibition of the NF‐kappaB pathway in intervertebral disc degeneration. Experimental Cell Research, 352(2), 322–332. 28215636 10.1016/j.yexcr.2017.02.023

[fsn32519-bib-0014] Liang, H. , Yang, X. , Liu, C. , Sun, Z. , & Wang, X. (2018). Effect of NF‐kB signaling pathway on the expression of MIF, TNF‐alpha, IL‐6 in the regulation of intervertebral disc degeneration. Journal of Musculoskeletal and Neuronal Interactions, 18(4), 551–556. 30511959 PMC6313038

[fsn32519-bib-0015] Liao, Z. , Wu, X. , Song, Y. , Luo, R. , Yin, H. , Zhan, S. , Li, S. , Wang, K. , Zhang, Y. , & Yang, C. (2019). Angiopoietin‐like protein 8 expression and association with extracellular matrix metabolism and inflammation during intervertebral disc degeneration. Journal of Cellular and Molecular Medicine, 23(8), 5737–5750. 10.1111/jcmm.14488 31211513 PMC6653761

[fsn32519-bib-0016] Lu, L. , Qin, A. , Huang, H. , Zhou, P. , Zhang, C. , Liu, N. , Li, S. , Wen, G. , Zhang, C. , Dong, W. , Wang, X. , Dou, Q. P. , & Liu, J. (2011). Shikonin extracted from medicinal Chinese herbs exerts anti‐inflammatory effect via proteasome inhibition. European Journal of Pharmacology, 658(2–3), 242–247. 10.1016/j.ejphar.2011.02.043 21392503 PMC3299007

[fsn32519-bib-0017] Lu, S. L. , Dang, G. H. , Deng, J. C. , Liu, H. Y. , Liu, B. , Yang, J. , Ma, X. L. , Miao, Y. T. , Jiang, C. T. , Xu, Q. B. , Wang, X. , & Feng, J. (2020). Shikonin attenuates hyperhomocysteinemia‐induced CD4(+) T cell inflammatory activation and atherosclerosis in ApoE(‐/‐) mice by metabolic suppression. Acta Pharmacologica Sinica, 41(1), 47–55. 10.1038/s41401-019-0308-7 31607752 PMC7468273

[fsn32519-bib-0018] Luo, R. , Song, Y. , Liao, Z. , Yin, H. , Zhan, S. , Wang, K. , Li, S. , Li, G. , Ma, L. , Lu, S. , Zhang, Y. , & Yang, C. (2019). Impaired calcium homeostasis via advanced glycation end products promotes apoptosis through endoplasmic reticulum stress in human nucleus pulposus cells and exacerbates intervertebral disc degeneration in rats. FEBS Journal, 286(21), 4356–4373. 10.1111/febs.14972 31230413

[fsn32519-bib-0019] Pfirrmann, C. W. , Metzdorf, A. , Zanetti, M. , Hodler, J. , & Boos, N. (2001). Magnetic resonance classification of lumbar intervertebral disc degeneration. Spine, 26(17), 1873‐1878. 11568697 10.1097/00007632-200109010-00011

[fsn32519-bib-0020] Risbud, M. V. , & Shapiro, I. M. (2014). Role of cytokines in intervertebral disc degeneration: Pain and disc content. Nature Reviews Rheumatology, 10(1), 44–56. 10.1038/nrrheum.2013.160 24166242 PMC4151534

[fsn32519-bib-0021] Ruiz‐Fernandez, C. , Francisco, V. , Pino, J. , Mera, A. , Gonzalez‐Gay, M. A. , Gomez, R. , Lago, F. , & Gualillo, O. (2019). Molecular relationships among obesity, inflammation and intervertebral disc degeneration: Are adipokines the common link? International Journal of Molecular Sciences, 20(8), 2030. 10.3390/ijms20082030 PMC6515363 31027158

[fsn32519-bib-0022] Shen, J. , Fang, J. , Hao, J. , Zhong, X. , Wang, D. , Ren, H. , & Hu, Z. (2016). SIRT1 inhibits the catabolic effect of IL‐1beta through TLR2/SIRT1/NF‐kappaB pathway in human degenerative nucleus pulposus cells. Pain Physician, 19(1), E215–E226. 26752489

[fsn32519-bib-0023] Tong, Y. , Bai, L. , Gong, R. , Chuan, J. , Duan, X. , & Zhu, Y. (2018). Shikonin protects pc12 cells against beta‐amyloid peptide‐induced cell injury through antioxidant and antiapoptotic activities. Scientific Reports, 8(1), 26. 29311595 10.1038/s41598-017-18058-7 PMC5758797

[fsn32519-bib-0024] Wang, F. , Mayca Pozo, F. , Tian, D. , Geng, X. , Yao, X. , Zhang, Y. , & Tang, J. (2020). Shikonin inhibits cancer through p21 upregulation and apoptosis induction. Frontiers in Pharmacology, 11, 861. 32581812 10.3389/fphar.2020.00861 PMC7296065

[fsn32519-bib-0025] Wang, S. Z. , Rui, Y. F. , Tan, Q. , & Wang, C. (2013). Enhancing intervertebral disc repair and regeneration through biology: Platelet‐rich plasma as an alternative strategy. Arthritis Research & Therapy, 15(5), 220. 10.1186/ar4353 24165687 PMC3978730

[fsn32519-bib-0026] Wang, Y. , Che, M. , Xin, J. , Zheng, Z. , Li, J. , & Zhang, S. (2020). The role of IL‐1beta and TNF‐alpha in intervertebral disc degeneration. Biomedicine & Pharmacotherapy, 131, 110660. 32853910 10.1016/j.biopha.2020.110660

[fsn32519-bib-0027] Yang, C. , Liu, P. , Wang, S. , Zhao, G. , Zhang, T. , Guo, S. , Jiang, K. , Wu, H. , & Deng, G. (2018). Shikonin exerts anti‐inflammatory effects in LPS‐induced mastitis by inhibiting NF‐kappaB signaling pathway. Biochemical and Biophysical Research Communications, 505(1), 1–6. 30224056 10.1016/j.bbrc.2018.08.198

[fsn32519-bib-0028] Yang, J. , Wang, Z. , & Chen, D. L. (2017). Shikonin ameliorates isoproterenol (ISO)‐induced myocardial damage through suppressing fibrosis, inflammation, apoptosis and ER stress. Biomedicine & Pharmacotherapy, 93, 1343–1357. 10.1016/j.biopha.2017.06.086 28753907

[fsn32519-bib-0029] Yi, W. , Wen, Y. , Tan, F. , Liu, X. , Lan, H. , Ye, H. , & Liu, B. (2019). Impact of NF‐kappaB pathway on the apoptosis‐inflammation‐autophagy crosstalk in human degenerative nucleus pulposus cells. Aging (Albany NY), 11(17), 7294–7306. 31518335 10.18632/aging.102266 PMC6756901

[fsn32519-bib-0030] Zhong, H. , Zhou, Z. , Guo, L. , Liu, F. , Zheng, B. , Bi, S. , & Tian, C. (2021). The miR‐623/CXCL12 axis inhibits LPS‐induced nucleus pulposus cell apoptosis and senescence. Mechanisms of Ageing and Development, 194, 111417. 10.1016/j.mad.2020.111417 33333129

